# Horizontal Augmentation of Chronic Mandibular Defects by the Guided Bone Regeneration Approach: A Randomized Study in Dogs

**DOI:** 10.3390/ma15010238

**Published:** 2021-12-29

**Authors:** Anton Friedmann, Stefan Fickl, Kai R. Fischer, Milad Dalloul, Werner Goetz, Frederic Kauffmann

**Affiliations:** 1 Department of Periodontology, Faculty of Health, School of Dentistry, Witten/Herdecke University, 58455 Witten, Germany; miladdalloul1@hotmail.com; 2 Department of Periodontology, University of Würzburg, 97070 Würzburg, Germany; stefanfickl@fickl-krug.de; 3 Private Office, 90762 Fürth, Germany; 4 Center of Dental Medicine, Clinic for Conservative Preventive Dentistry, Division for Periodontology & Peri-Implant Diseases, University of Zurich, 8032 Zurich, Switzerland; kai.r.fischer@zzm.uzh.ch; 5 Private Office, 56727 Koblenz, Germany; 6 Department for Orthodontics, Friedrich-Wilhelm-University Bonn, 53111 Bonn, Germany; wgoetz@uni-bonn.de; 7 Private Office, 40210 Düsseldorf, Germany; kauffmann@kieferchirurgie.org

**Keywords:** lateral augmentation, histomorphometry, collagen membranes, degradation profile, bone substitutes, guided bone regeneration (GBR), animal study

## Abstract

Various biomaterial combinations have been studied focusing on their ability to stabilize blood clots and maintain space under soft tissue to support new bone formation. A popular combination is Deproteinized Bovine Bone Mineral (DBBM) placed with a native collagen membrane (NCM) tacked to native bone. In this study, we compared the outcome of this treatment option to those achieved with three different graft/membrane combinations with respect to total newly occupied area and the mineralized compound inside. After bi-lateral extraction of two mandibular premolars in five adult beagles L-shaped alveolar defects were created. A total of 20 defects healed for 6 weeks resulting in chronic type bone defects. At baseline, four options were randomly allocated to five defects each: a. DBBM + NCM with a four-pin fixation across the ridge; b. DBBM + RCLC (ribose cross-linked collagen membrane); c. DBBM + NPPM (native porcine pericardium membrane); and d. Ca-sulfate (CS) + RCLC membrane. Membranes in b/c/d were not fixed; complete tensionless wound closure was achieved by CAF. Termination after 3 months and sampling followed, and non-decalcified processing and toluidine blue staining were applied. Microscopic images obtained at standardized magnification were histomorphometrically assessed by ImageJ software (NIH). An ANOVA post hoc test was applied; histomorphometric data are presented in this paper as medians and interquartile ranges (IRs). All sites healed uneventfully, all sites were sampled and block separation followed before Technovit embedding. Two central sections per block for each group were included. Two of five specimen were lost due to processing error and were excluded from group b. New bone area was significantly greater for option b. compared to a. (*p* = 0.001), c. (*p* = 0.002), and d. (*p* = 0.046). Residual non-bone graft area was significantly less for option d. compared to a. (*p* = 0.026) or c. (*p* = 0.021). We conclude that collagen membranes with a prolonged resorption/barrier profile combined with bone substitutes featuring different degradation profiles sufficiently support new bone formation. Tacking strategy/membrane fixation appears redundant when using these biomaterials.

## 1. Introduction

Several types of biomaterials, such as bone substitutes, membrane barriers, and recently biologics are suggested to effectively support new bone formation in a process termed Guided Bone Regeneration (GBR). Various bone substitutes share the ability to stabilize blood clots and maintain space under a cell’s occlusive membrane barrier, as demonstrated by numerous animal trials and corroborated by human histologic observations [1,2,3,4,5]. The combination of a bone substitute with a membrane barrier, which is supposed to seclude the augmented area from the surrounding soft tissue is considered the core principle of GBR [3,6,7]. The crucial component for the bone formation process among the biomaterials applied in combinations is not yet determined. Particularly, the impact of the membrane barrier presence and of its longevity on bone formation is debated [8,9,10,11]. 

Omar et al. reviewed the membranes proposed for Guided Bone Regeneration (GBR) classifying them according to the type of biomaterials [12]. Xenogeneic collagens represent a group of highly biocompatible resorbable membranes with low immunogenicity but easy incorporation into the tissues [13,14]. In studies related to GBR, the literature reveals the highest frequency for a combination of deproteinized bovine bone mineral (DBBM) with a native non-cross linked collagen membrane (NCM) [15,16,17]. Recently proposed treatment protocols using this combination recommend four-point fixation tacking the membrane to the alveolar bone aiming at additional stabilization of the graft and the membrane [18,19,20,21]. Despite this dominance, in other studies, biomaterials applied in different combinations had similar success in supporting new bone formation [22,23,24]. 

The heterogeneity of xenogeneic collagen membranes is characterized by the degree of collagen cross-linking. On one hand, native collagen membranes can feature different degrees of crosslinking dependent on their individual tissue origin. On the other hand, collagen crosslinking can be increased by the manufacturing process. Accounting for the degradation profile encoded by the degree of cross-linking, the variety of collagen membranes resembles the diversity in bone substitutes taken by their degradation profiles [25]. The most intriguing question concerns the longevity of the membrane applied for covering the grafted zone beneath the soft tissue and its behavior in the case of soft tissue dehiscence [26]. The other underestimated target is the choice between a long-lasting membrane vs. a membrane with a short barrier function combined with bone substitutes of different origin and degradation profile [26,27,28,29,30]. 

To evaluate the impact on the longevity of membrane and type of bone graft materials, we conducted an animal trial. In this animal trial, we histomorphometrically assessed the outcomes of the horizontal augmentation procedure performed following the GBR principles and using four different material combinations with differing resorption profiles randomly allocated to 20 chronic mandibular L-shaped defects. The primary outcome is the area extension of newly formed bone within the area of interest, while the secondary parameter is the change in the alveolar ridge width assessed in the horizontal dimension at the most crestal level and at three further levels apical to the crest.

## 2. Materials and Methods

The study protocol was approved by the Directorate of Foodchain Security and Animal Health Care Pest County Government Office, Hungary (file number: PEI/001/961-1/2013). The study was carried out at the Research Institute for Animal Breeding and Nutrition, Herceghalom/Hungary. 

Five adult male beagle dogs at least 12 months old and weighing an average of 8.5–11.5 kg were used. The dogs were kept in pairs in kennels of 5 m^2^ with straw bedding and elevated wooden resting places and were walked twice daily for at least 30 min. Animals were fed commercially available dog food (Bonafarm, Nagyigmánd, Hungary), and water was provided ad libitum. Surgeries were carried out under intravenous administration of ketamine hydrochloride (2.5 mL/10 kg; Ketavet 10%, Pfizer, Berlin, Germany) and xylazine hydrochloride (1 mL/10 kg; Xylavet 2%, Sanofi-Aventis, Budapest, Hungary) every 15 min for general anesthesia. Metamizole (1 mL/10 kg, Pfizer, Berlin, Germany) was administered intramuscularly for 3 days for pain control and Amoxicillin hydrochloride (150 mg, 1 mL/10 kg, Pfizer, Berlin, Germany) was injected intramuscularly for infection prevention.

**Figure 1:**
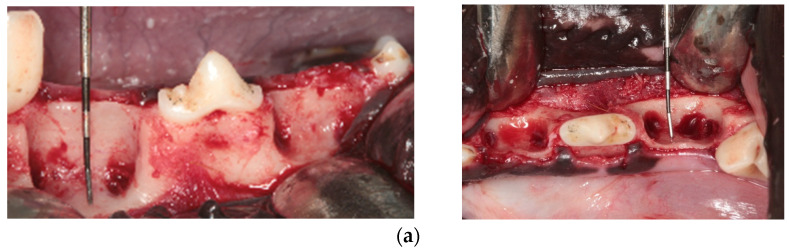

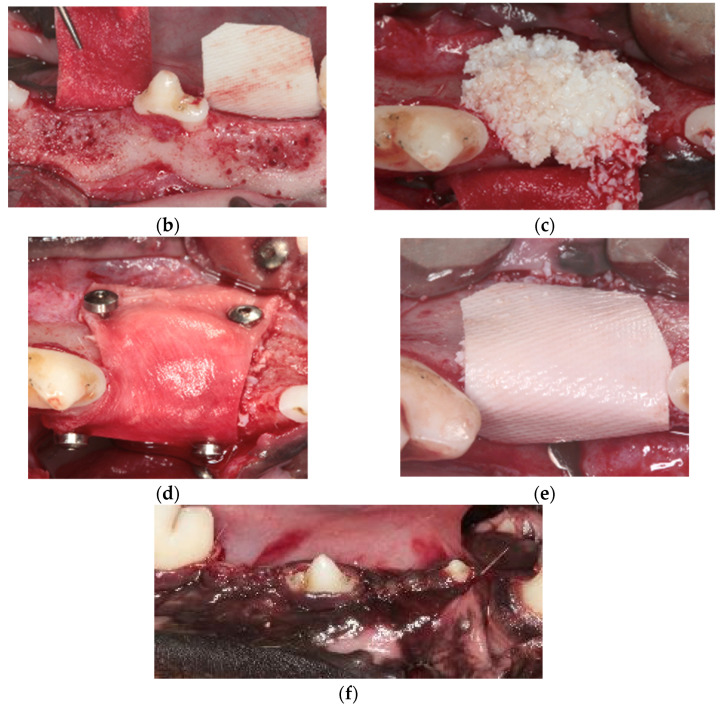
Images of clinical documentation of defect configuration and GBR procedures. (**a**). Standardization of surgically created defects measured by the means of periodontal probe following tooth removal at the first surgery. (**b**). The positioning of the membranes for option a. and option b. after the cortical perforations in the buccal bone are completed at the second surgery. (**c**). The bone substitute (DBBM) in place. (**d**). The four-point membrane tacking by titanium tacks for option (**a**). (NCM). (**e**). The non-fixated membrane adaptation for option (**b**). (RCLC). (**f**). The complete tensionless soft tissue closure.

The surgical procedures included an extraction of the mandibular premolars P2 and P4 and surgical grinding of alveolar bone to an L-shaped buccal bone defect with an extension of approximately 6–7 mm × 8–10 mm × 2.5–3 mm (corono-apical × mesio-distal × facio-oral). Rotating instruments were used and the soft tissue was sutured for defect closure [31] (Figure 1a). The second, regenerative surgery followed 6 weeks later. All sites displayed a chronic type of alveolar bone deficiency with an almost completely remodeled crestal plate. The cortical surface of the defects was perforated by round bur to achieve bleeding from the bone marrow before application of the bone substitute [32] (Figure 1b). A non-involved investigator carried out the random allocation of treatments and being present at surgery, disclosed the material combination site by site for the surgeons. Thus, the team of surgeons (A.F., S.F., F.K., K.R.F. and M.D.) learned the treatment modality after the full-thickness tissue flap was reflected, and the chronic bone defect was denudated. Four treatment options (a.–d.) were randomly allocated to an equal number of defects. Option a. received deproteinized bovine bone mineral and a non-cross-linked native collagen membrane (DBBM + NCM; BioOss and Biogide, Geistlich, Switzerland) applied with a four-pin fixation, two on the buccal and two on the lingual aspect of the ridge (Figure 1c,d). Option b. was treated with DBBM and a ribose cross-linked collagen membrane (DBBM + RCLC; BioOss, Geistlich, Switzerland and Ossix^®^Plus, Regedent, Germany). Option c. received DBBM and a native porcine pericardium membrane (DBBM + NPPM; BioOss, Geistlich, Switzerland and Smartbrane, Regedent, Germany). Option d. received Ca-sulfate and an RCLC membrane (CS + RCLC; 3DBond and Ossix^®^Plus, Regedent, Germany). Distinct from option a., membranes in options b., c., and d. received no additional fixation but the stabilization by the soft tissue flap itself (Figure 1e). After completion of the second surgery, all sites were closed by using the CAF technique for coronal advancement of the soft tissue achieving tensionless suture by resorbable suture material (Monocryl, 5.0, Ethicon, Germany) (Figure 1f).

The healing was allowed for 3 months; thereafter, termination by pentobarbital injection followed. The mandibles were completely retrieved and fixated in 4% formaldehyde for several days, lasting through transportation from the surgical facility to the lab facility at University of Bonn. The mandibles were first placed into a µCT and afterwards sectioned into blocks with a diamond saw in a frontal (bucco-lingual) orientation. The specimen underwent non-decalcified processing.

### Histologic Procedure

After fixation, the samples were dehydrated in an ascending ethanol series from 70% up to 100% for 9 weeks, and infiltrated with ultraviolet light activated polymethylmetacrylate (PMMA, Technovit^®^ 721100, Heraeus Kulzer, Hanau, Germany) for 5 weeks. Parallel sections were then cut from the specimens using a microsaw device (EXAKT Advanced Technologies, Norderstedt, Germany) in the vestibulo-lingual direction and ground up to 20 μm thickness using a microgrinding system (EXAKT). Sections were stained using toluidine blue or paragon staining without the removal of the plastic medium. All biopsies were cut and ground in serial sections. Two central sections per block in each group were included in analysis. All ground sections were photographed (1:1) with a reflecting microscope (Leica, Wetzlar, Germany) using a Leica camera DFC420 with software V3.8 (Leica) and evaluated under a light microscope (Zeiss-Axio-Imager^®^, Zeiss, Jena, Germany) at original magnifications between 6.5× and 50×. Three investigators (A.F., F.K. and W.G.) determined the region of interest (ROI) representing the augmented area and considered enclosing the area occupied by newly formed tissue within the cross-section only. The parameters for microscopic analysis were the total ROI of newly formed tissue and the area occupied by non-bone integrated residual graft particulate inside the total ROI. The delta between total ROI and non-bone graft residues was calculated. The histomorphometric assessment followed, using Image J software (NIH) in standardized magnification at 6.5-fold. For statistical analysis, we performed a QQ plot paired with the results of a Shapiro–Wilk normality test for data distribution. Thereafter, ANOVA and post hoc test for Bonferroni correction were carried out. The level of statistical significance was set at *p* < 0.05.

**Figure 2:**
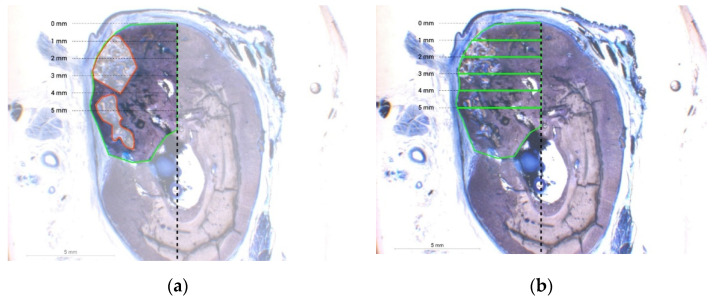
The microscopic image for option **a.** (DBBM + NCM) in toluidine blue stain (×6.5). (**a**). The total ROI (green box) and highlighted non-bone graft area (red box). (**b**). The schematic position of the perpendicular and five highlighted levels for assessment of the horizontal ridge dimension.

To assess the secondary outcome, a fictitious perpendicular was dropped at the lingual cortical wall in every section as a reference level for the horizontal extension towards the buccal edge of the ridge. The width from the perpendicular to the edge of the ridge was calculated at the crestal level and at 1, 3 and 5 mm distance apically to this level (Figure 2a,b) [33].

## 3. Results

All sites healed uneventfully; a total of 20 samples were retrieved and processed. However, within group b., two out of five specimens displayed obvious signs of sample distortion in the sections and were excluded from analysis. Thus, the calculation of the primary and secondary parameters was based on n = 5 in the treatment options a., c., and d. and on n = 3 for option b.

**Figure 3:**
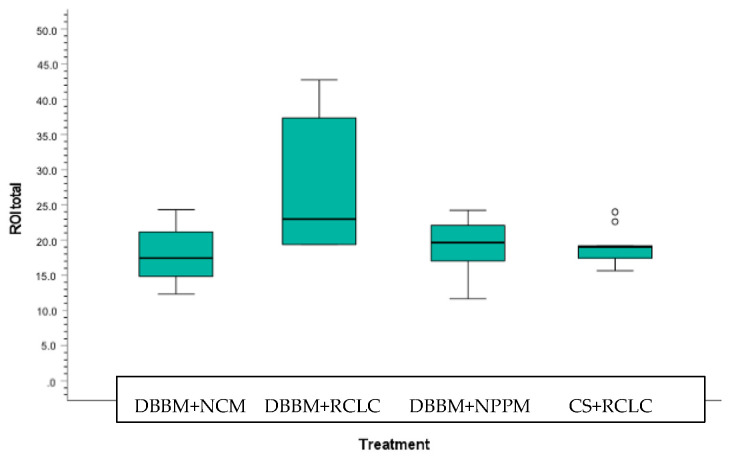
Box and whisker plot of the total ROI distribution across the four treatment options.

**Figure 4:**
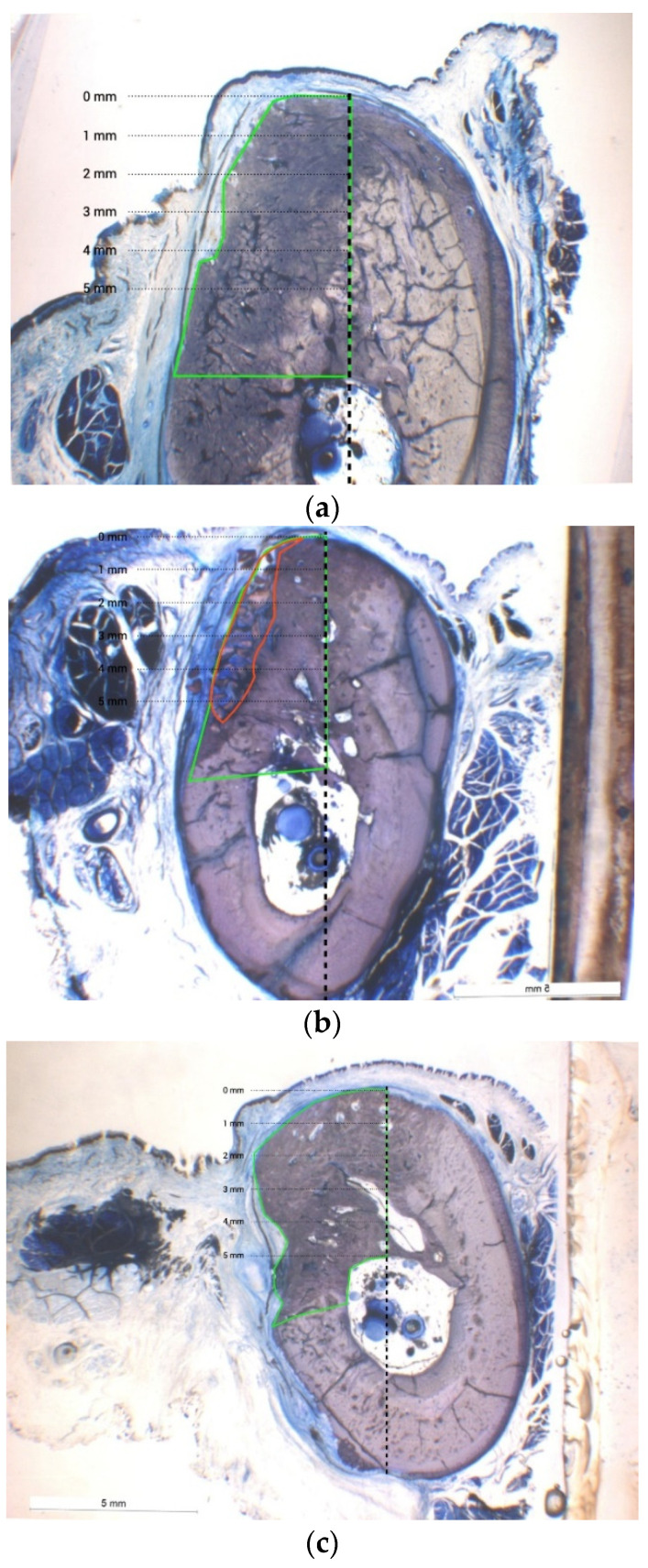
The microscopic images for options b., c., and d. in toluidine blue stain (×6.5). (**a**) Total ROI (green box) and absent non-bone graft area (red box) for option b. (DBBM + RCLC). (**b**) Total ROI (green box) and highlighted non-bone graft area (red box) for option c. (DBBM + NPPM). (**c**) Total ROI (green box) and absent non-bone graft area (absent red box) for option d. (CS + RCLC).

Despite the diminished number of samples included, the total newly formed tissue area extension was significantly greater for option b. treated with DBBM + RCLC (27.48 ± 4.13 mm^2^) when compared to a. treated with DBBM + NCM (17.83 ± 1.26 mm^2^), c. treated with DBBM + NPPM (19.04 ± 1.20 mm^2^), or d. treated with a resorbable CS + RCLC (19.14 ± 2.51 mm^2^). The differences for b. vs. a., b. vs. c., and b. vs. d. were statistically significant (*p* = 0.006; 0.019; 0.021, respectively). Treatment option b. resulted in the largest newly formed tissue area showing the most complete incorporation of the DBBM particulate material into the newly mineralized portion of the sample. Option c. revealed the second largest new tissue area extension. Option d. displayed similar area extension for newly formed bone as option c.; however, option d. showed almost no residues of the particulate CS graft material (Figure 3: the box and whisker plot of the total ROI graphically represents a large number of descriptive parameters). Thus, the residual non-bone graft area was significantly less for option d. vs. a. or c. (*p* = 0.026 and 0.021, respectively). Table 1 displays the histomorphometrical medians and interquartile ranges (IRs). The new tissue area extension (total ROI) for option c. using the pericardium membrane (NPPM) was notably greater compared to options a. and d., but without being statistically significant. The amount of residual non-bone integrated residual graft was similar for options a. and c. (Figure 2 and Figure 4).

**Figure 5:**
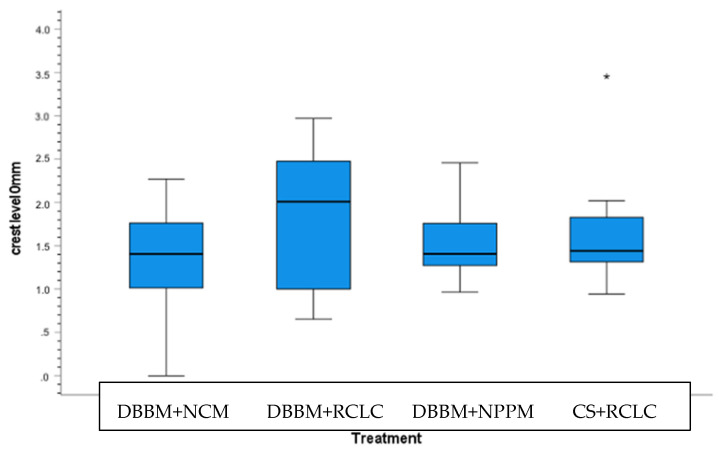
Box and whisker plot of the change in horizontal thickness at the crestal level (level 0).

The change in the ridge width yielded non-significant differences between treatment options at all four horizontal levels. However, at the crestal level, the median for option b. was 2.01 mm (interquartile range (IR): 1.00/2.47), indicating a greater tendency for an increased ridge width compared to option a. (1.40 mm; IR: 1.02/1.76), option c. (1.40 mm; IR: 1.27/1.76), and option d. (1.43 mm; IR: 1.25/1.93) (Figure 5) At further apical levels, the values for the achieved alveolar ridge width were comparable between all treatment options (Table 2, Figure 2a,b and Figure 3). 

## 4. Discussion

The study was conducted to evaluate the potential of different biomaterial combinations to support new bone formation and to estimate the quality and quantity of newly formed tissues in a chronic type of alveolar ridge defect applying the GBR principle. Therefore, non-decalcified processing of the specimen was considered suitable to maintain the tissue composition and the content of the biopsy regarding the particulate graft material at its best. We made efforts to carefully separate the blocks and to initiate the embedding process. Nevertheless, the comparison between the µCT images which were obtained before separating the mandibles and the microscopic images, indicates that deformation must have occurred after the µCT procedure in two blocks, both accounting for option b. specimens. Thus, the exclusion of the two option b. specimens from the histomorphometric analysis is a result of technical problems during the laboratory processing. 

Nevertheless, the results for option b. based on a diminished number of specimens valid for assessment, showed the greatest extension of the newly formed tissue (total ROI) accompanied by the smallest range in residual non-bone integrated bone substitute particles. The small delta between ROI and the none-bone graft area indicated that the assessed area mainly resulted in mineralized tissue compound, i.e., new bone with enclosed portion of non-resorbing particulate. 

A comparison of results for options a., c., and d. did not yield statistically significant differences. However, the combination of DBBM with a native porcine pericardium membrane (NPPM, option c.) achieved the second largest amount of newly formed tissue area with fewer non-bone integrated particles than a. Similar total ROI extension was estimated for option d. The bone substitute used for option d. was calcium-sulfate (CS), a fast-resorbing material [34], which, according to the microscopic analysis at higher magnification, was almost completely absent at the total ROI level in the specimens after the three month period. We can hypothesize that the extension of newly formed bone within the total ROI area benefited from the fact that no residual graft remained enclosed when compared to similarly extend total ROI area assessed for option b. 

The findings for option a. yielded significantly less bone formation under the tacked native collagen membrane vs. other options, pointing out that membrane longevity may contribute to bone formation rather than membrane fixation. Thus, the comparison of resorbable synthetic membranes vs. native collagen membranes used in a canine model with the DFDB particulate revealed significantly diminished bone fill in sites treated with the native collagen membrane [11]. The residual graft particulate appeared embedded in connective tissue, which did not show signs of ongoing ossification at a greater magnification. 

In our study, treatment option a. was associated with the greatest extension of the non-bone graft content among all four options. The obtained results indicate a strong correlation between membrane resorption/barrier profile and the amount of newly created bone. Among tested membranes, the ribose/sugar-crosslinked membrane (RCLC) features the greatest longevity, followed by the native porcine pericardium membrane (NPPM). Native collagen membranes (NCM) are known for their fast resorption pattern. Hence, in a GBR animal model the NCM resorbed significantly faster compared to NPPM (NCM: 4–8 weeks vs. NPPM 8–12 weeks, respectively) [34]. RCLC is known to resorb within 4–6 months [13] and appeared almost completely maintained within the recipient tissues after a 26 week period in rats [35]. Accordingly, long lasting collagen membranes, such as NPPM and particularly RCLC appear to promote new bone formation within the bone defect rather than fast resorbing NCM. Beyond this observation, the results point out that tack fixation of a fast resorbable collagen membrane does not necessarily result in greater volume of augmented tissues as achieved with long lasting collagen membranes used without tacking.

During assessment of the secondary parameter, the difference in the ridge width at four vertical levels revealed non-significant differences between all treatment groups. This finding does not contradict the results discussed above. In fact, it confirms that the calculated change in width at a singular horizontal level does not reflect true gain in newly formed bone. Histologies by Thoma et al. showed similar results in terms of achieved tissue volume. Qualitative analysis, however, revealed particulate bone substitute embedded in connective tissue instead of becoming part of newly formed bone. Related to the first bone-to-implant contact (fBic) assessed at the buccal aspect, the data clearly demonstrated that for the placement of dental implants, the tissue embedded graft particles were inferior compared to the group in which “soft bone” was removed and an additional augmentation was carried out [36]. 

Our observations corroborate data reported by other authors, who only investigated the tissue volume change as a primary goal. In agreement with this, a 5 year comparison of simultaneous augmentation around implants performed with a non-resorbable PTFE vs. a resorbable native collagen membrane (NCM) revealed negligible profilometric changes in the ridge profile between both groups. However, the reduction in buccal bone thickness was significantly higher in the group treated with NCM [26]. Apparently, in this group, soft tissue enhancement in the area compensated for the bone reduction at the 5 year observation. The future clinical impact of such tissue alteration is yet to be mapped out.

In this report, we abstained from presenting µCT calculations. Since unseparated mandibles were placed into the µCT-radiograph, the obtained images did not correspond with the precise cross-sectioning of the blocks strictly perpendicular to the edentulous area augmented. The error in deterioration between the two imaging techniques is inaccessible and therefore we omitted the analysis of µCT-based calculations. Moreover, the rationale behind an X-ray- based evaluation (CBCT, µCT, others) of defect areas augmented with slow or non-resorbing bone grafts remains questionable considering the histologic finding of the DBBM particles embedded in granulation tissue in some groups.

## 5. Conclusions

Histomorphometric results favor option b. as being the most efficient in promoting new bone formation and integrating bone substitute into newly formed bone. Option c. fares second regarding these parameters, followed by option d. which used RCLC combined with a resorbable CS. Within this study, the pin fixation for option a. with the DBBM + NCM combination did not reveal additional benefit. The sugar cross-linked collagen membrane in combination with DBBM provided a superior outcome, indicating the impact of membrane barrier in bone augmentation. However, further animal studies are required to substantiate these findings.

## Figures and Tables

**Table 1:** The mean values, Standard of Error (SE), and *p*-values for the ROI, the extension of non-bone graft area within the ROI, and the delta between the ROI and non-bone graft area. The statistically significant differences resulting from group-by-group comparison are indicated by *; ^‡^; ^¥^.

Parameter	a. DBBM + NCM	b. DBBM + RCLC	c. DBBM + NPPM	d. CS + RCLC
ROI total mean ± SE	17.83 ± 1.26 *	27.48 ± 4.13 * ^‡ ¥^	19.04 ± 1.20 ^‡^	19.14 ± 2.51 ^¥^
*p*-values	* b. vs. a. 0.006 ^‡^ b. vs. c. 0.019 ^¥^ b. vs. d. 0.021			
Residual non-bone graft mean ± SE	3.08 ± 1.03 *	0.72 ± 1.27	2.64 ± 0.83 ^‡^	0.03 ± 0.03 *^,‡^
*p*-values	* a. vs. d. 0.026 ^‡^ c. vs. d. 0.021			
∆ ROI-none bone graft mean ± SE	14.76 ± 3.94 *	26.76 ± 10.62 *^,‡,¥^	15.89 ± 3.47 ^‡^	19.11 ± 2.52 ^¥^
*p*-values	* b. vs. a. 0.001 ^‡^ b. vs. c. 0.002 ^¥^ b. vs. d. 0.046			

**Table 2:** The medians and IRs for the horizontal thickness change at four determined levels (crestal, −1 mm, −3 mm, −5 mm; all differences non-significant, n.s.).

Horizontal Level	a. DBBM + NCM	b. DBBM + RCLC	c. DBBM + NPPM	d. CS + RCLC
Level 0	1.405 1.01/1.76	2.10 1.00/2.48	1.40 1.72/1.76	1.44 1.31/1.83
Level 1	2.90 1.71/3.01	2.38 1.90/3.71	2.86 1.90/3.49	3.06 2.41/3.33
Level 2	2.95 2.46/3.55	2.94 2.79/3.50	3.26 2.61/3.84	3.26 2.61/3.76
Level 3	3.23 2.74/3.65	3.56 3.54/3.61	3.05 2.72/4.02	3.56 3.37/3.48

## Data Availability

The data presented in this study are available upon request from the corresponding author.
